# Do algorithm traders mitigate insider trading profits?: Evidence from the Thai stock market

**DOI:** 10.1371/journal.pone.0255057

**Published:** 2021-07-26

**Authors:** Nopparat Wongsinhirun, Pattanaporn Chatjuthamard, Sirimon Treepongkaruna, Tanakorn Likitapiwatc

**Affiliations:** 1 Sasin School of Management, Chulalongkorn University, Bankok, Thailand; 2 Center of Excellence in Management Research for Corporate Governance and Behavioural Finance Sasin School of Management, Chulalongkorn University, Bangkok, Thailand; 3 UWA Business School, The University Western Australia, Crawley, WA, Australia; 4 Chula Business School, Chulalongkorn University, Bangkok, Thailand; University of Almeria, SPAIN

## Abstract

This paper asks whether algorithm traders (AT) mitigate insider trading profits in the Thai stock market over the period of 2010–2016. We find that in general it does but not in the case of buy side, big trades nor the executive trades. Our findings suggest that, to some extent, AT can take important role to increase an efficiency in stock market by processing the public information and incorporating it into price at ultra-fast speed. Additional robustness checks based on the instrumental variable approach confirm our findings.

## Introduction

Corporate insider trading performance has been studied extensively over the past 60 years. Numerous studies document abnormal stock returns earned by corporate insiders [1–5]. Trading profits earned by corporate insiders are evidence against strong form market efficiency. Recently, several studies such as Hendershott et.al. [6], Brogaard et al. [7], Martinez and Rosu [8], Foucault et al. [9] and Boehmer et al. [10] suggest algorithmic traders with their ability to process all publicly available information at ultra-high speed improve liquidity and information efficiency. The algorithmic trading in the Stock Exchange of Thailand (SET) is still in its early stage. Likitapiwat [11] documents the maximum proportion of AT activities relative to the entire market is 13.25% by number of trades and 4% by volume in 2011. Given the increasing role of AT in the Thai stock market, this paper explores whether AT can improve market efficiency by mitigating insider trading profits in the Thai stock market.

Prior studies find AT improves liquidity and information efficiency. Hendershott et al. [6] document algorithmic trading improves liquidity in the NYSE while Brogaard et al. [7] suggest that AT enhances price efficiency. Martinez and Rosu [8] and Foucault et al. [9] document the advantage of algorithmic trading, stating that its speed enables every transaction to transmit and incorporate public information more quickly into prices. In addition, Jovanovic and Menkveld [12], Martinez and Rosu [8], and Chakrabarty et al. [13] support that AT can enhance market efficiency. In stock markets where liquidity condition and information environment are being increasingly shaped by algorithmic traders, AT could potentially become mechanism to discipline the behavior of corporate insiders.

Based on a sample of 6.156 stock-month insider transactions for 409 stocks across 7 years, our empirical evidence shows that in general algorithmic trading reduces corporate insider trading profit but not in the case of buy side, big trades nor the executive trades. Additional robustness checks using the 2SLS instrumental variable approach, different event windows and different proxies for AT confirm our findings.

Our paper contributes to literature in the following ways. First, we contribute to the corporate insider trading literature in an attempt to identify potential additional determinants of insider trading profits. In the same spirit with To et al. [14], we explore the information asymmetry between executive and non-executive insiders [15–18]. We provide new evidence that algorithmic traders do effect insiders’ ability to extract rent on their private information for small to medium trade size. However, the true insiders being executive directors continue to extract rent from their private information.

Second, we contribute to literature on algorithmic trading and market efficiency. Hendershott et al. [6] suggest AT improves liquidity while Brogaard et al. [7], Jovanovic and Menkveld [12], Martinez and Rosu [8], and Chakrabarty et al. [13] provide evidence that AT enhances market efficiency. We compliment these prior studies by presenting that in the presence of AT, the insider trading profits are lower for the insider sales with small to medium trade size.

Finally, we contribute to the literature on informed traders competing for information. Massa et al. [19] explore two groups of informed traders: short sellers vs. corporate insiders and find insiders trader more and faster when short sellers are presented. Similarly, To et al. [14] demonstrate that short sellers can affect insider trading profit. Huang et al. [20] document that algorithmic traders reduce insider trading profits in the U.S market. We extend these previous works by showing that algorithmic traders (another type of informed traders) do affect insider trading profits in the emerging market.

The remainder of this paper is organized as follows. Section 2 reviews related literature and develops hypothesis. Section 3 describes data and methods. Section 4 presents empirical results and section 5 concludes.

## Review of related literature and hypothesis development

### A. Corporate insiders trading

Corporate insiders trade on privileged information about the firms and earn positive (negative) abnormal returns for the buy (sell) transactions [1, 21–24]. A number of studies attempt to explain reasons behind insider trades (purchases vs. sales). It is often argued that insider purchases are more informative and more likely to occur when insiders observe price sensitive information while insider sales are often attributed to non-informational reasons such as liquidity reasons to unwind directors’ stock-based compensation. Studies supporting this argument include Lakonishok and Lee [25] and Jeng et al. [26] documenting that the buy trades of insiders are more informative than sell trades; and Ofek and Yermack [27] and Meulbroek [28] providing evidence that insiders sell for liquidity reason.

Another strand of research in corporate insider distinguishes between different types of insiders based on the information hierarchy hypothesis. Studies supporting information hierarchy hypothesis include Seyhun [23], Lin and Howe [29] and To et al. [14] documenting cumulative abnormal returns following the transactions by executives are significantly higher than those by non-executive directors; Masson and Madhavan [30] reporting lower firm performance when executives actively use insider information in their stock trading; and Aboody et al. [31] providing evidence that executives rely on private information in their sales.

### B. Algorithmic trading and information efficiency

AT is another group of informed traders and to some extent can help improve market efficiency by incorporating information into price at the ultra-high speed. Prior studies supporting this argument include Zhang [32] and Chakrabarty et al. [13] documenting that high frequency traders (HFT) are particularly skilled in incorporating hard information; Carrion [33], Hirschey [34], and Brogaard et al. [7] demonstrating that HFT trades can forecast price changes in several seconds ahead and are more likely to have permanent price effects; and Foucault et al. [9] proposing a mechanism by which HFT directly contributes to information gathering by trading on the Brownian news surprises.

### C. Hypotheses development

Corporate insiders trade on private information about the firm and report their trading activities with a delay. In Thailand, the SEC requires all insiders to report their transactions within three days after the trading day and the trading information is shown on the SEC website at the end of the day three. On contrary, algorithmic traders trade on all available public information at the ultra-high speed as such their information gathering are short-horizon comparing to corporate insiders. Given the nature of long horizon and delay in reporting of insider trades, it is unlikely that AT could detect insider trades. As noted by Hendershott et al. [6], Brogaard et al. [7] and Boehmer et al. [10], algorithmic trading clearly indicates that AT improves liquidity and informational efficiency. This implies that the presence of AT makes market to be more liquid, allowing one to trade a small quantity at a lower spread or a more significant amount with less price impact. Theoretically, the presence of AT could either enhance or reduce insider trading profits.

According to asymmetric information hypothesis, the higher the information asymmetry, the higher the insider trading profit [35]. The reason is that market makers increase the bid-ask spread to compensate for the risk of trading with an insider who trades with high level of private information. As algorithmic traders reduce degree of information asymmetry in the market, their presence could lower insider trading profits.

On the other hand, attentive insider trading hypothesis proposed by Alldredge and Cicero [36] suggest that corporate insiders are actually the most attentive to the holdings of their own stocks, motivated them to recognize any profitable trading opportunities from the public information. Hence, corporate insiders could take advantage of more liquid and efficient market induced by AT and make profitable trading from the public information. This predicts a higher insider trading profit in presence of the AT. We postulate our first hypothesis as follows:

*H1: There is no relation between algorithmic trading (AT) and insider trading profits*.

In addition, trade size can influence both corporate insider trading and AT performance. Corporate insiders who have private information prefer to trade large amounts of shares at any given price [37, 38]. Larger trade size usually has more pronounced price impact. However, larger trade size benefits high-frequency trading [39] and HFTs face lower adverse selection costs than non-HFTs when supplying liquidity in larger trades [33]. Hence, it is likely that trade size could influence the relation between AT and insider trading profits. This leads us to our second hypothesis as follows:

*H2: Trade size has no influence on the relation between algorithmic trading (AT) and insider trading profits*.

Finally, information hierarchy can also affect the relation between AT and corporate insider trading profit. According to information hierarchy hypothesis, executives have better access to private information than non-executives do [14, 23, 29]. Hence, executives are likely to be true insiders and their trading profits may not be affected by the presence of AT. However, AT could potentially affect trading profits by non-executives. We postulate our last hypothesis as follows:

*H3: There is no relation between algorithmic trading (AT) and executives (or non-executives) trading profits*.

## Data and summary statistics

### A. Data

Two sets of data are obtained for this study. First, the algorithmic trading data, computed by using tick data provided by the Stock Exchange of Thailand. Following existing literature, we use two proxies for AT as follows. The ratio of the number of quoted messages to total value trading is called “AT Proxy1” while the ratio of the total volume traded to total volume across all orders is called “AT Proxy2”. All data are collected over the period of January 1, 2010 to December 31, 2016.

Second, the Thai insider trading data is obtained from Thompson Reuters Insider Database. The data is accessible over the period of 2010–2016. We extract detailed information on insider transactions, including personal identification, transaction date, stock, traded volume and whether it is a buy or sell. This database contains legal trading of all board of directors and management reporting in their purchase or sale of their stocks conformed to the rules set forth by the Securities and Exchange Commission (SEC). In every transaction in this report, we count as an event and calculate the cumulated abnormal returns (CARs), using daily stock return and return on the SET100 as a proxy for market returns. The returns on stocks and SET100 are obtained from the Datastream. The final sample includes 6,156 stock-month insider transactions for 409 stocks. All data are collected over the period of January 1, 2010 to December 31, 2016.

### B. Construction of variables

Two proxies are adapted to represent AT intensity. First, we follow the procedure detailed in Hendershott et al. [6] and Boehmer et al. [40] to construct the AT proxy. Specifically, using the order-level information, we count the number of electronic messages sent by traders to market centers (including order entry, amendment, cancellation, etc.) using the raw message traffic numbers, but there has been an increase in trading volume over the same interval. Without normalization, a raw message traffic measure may just capture the increase in trading rather than the change in the nature of trading. Therefore, for each stock in each month, we calculate our daily AT, as the number of electronic messages per of trading volume (AT Proxy1) as follows:

ATProxy1=(numberofmessages/Tradingvolume) (1)

The second AT proxy is adapted from Weller [41], trade-to-order volume ratio. Trade volume is the number of shares traded. Order volume is the volume of total orders placed. The trade-to-order volume ratio is one of several measures designed to characterize the nature of order placement and cancellation in equity markets. This ratio is interpreted reversely to the first proxy since the higher ratio means the lower AT intensity. To ease the interpretation of both AT proxies, we inverse the formula to be order-to-trade ratio as follows:

ATProxy2=(Totalorderedvolume/Totaltradedvolume) (2)

Next, we obtain our monthly change in AT from the change in AT during two adjacent months as follows:

$$AT\;proxy={AT}_{t}-{AT}_{t-1}$$ (3)

Another key variable of interests is insider trading profits, proxied by cumulative abnormal return based on an event study method. We expect a positive (negative) CAR for purchase (sale) transactions represents a profit. For this reason, the daily abnormal stock returns of insider trades are estimated for purchase and sale separately. The event date (t_0_) is taken to be the date of the insider trade, and an event period is spanning 20 days in the interval (t_1_ to t_20_). Abnormal returns are generated from the market model, where ARi,j,t is the risk-adjusted abnormal returns of firm i at day t of transaction j, Ri,t is the return on firm i at day t and Rm,t is the corresponding return on the market index (SET100) at time t. The estimation period to estimate the market model parameters is (t_-50_ to t_-5_):

$${AR}_{i,j,t}=R_{i,i,t}-\alpha_{i,j}-\beta_{i,j}{Rm}_{t}$$ (4)

To calculate insider profit, we calculate CAR of each insider transaction j by cumulating ARs over the event window of n = 20 days (1 month) after the transaction. As our sample is at monthly interval, we focus on the cumulative return over the 20 trading day window. For robustness checks, we also compute the cumulative return over one week and 3 month windows (e.g. CAR(1,5) and CAR(1,60)) and include them in our baseline regression, reported in Table 2.


$${CAR}_{i,j}=\sum_{n=1}^{20}{AR}_{i,j,n}$$ (5)

We then average CAR at stock-month level for purchase and sale transactions separately. Following the insider trading literature, we also include a number of control variables for the firm-specific characteristics such as firm size, leverage, return on assets and the book-to-market ratio, all taken from previous year end. To control the momentum effect in stock returns, we include the CAR over the 1-year window prior to a given insider transaction. Finally, we also control for insider trading characteristics by including the average trade size [32, 42]. In same spirit of To et al. [14], we classify insider level into 8 levels and separate this list into two types of insider levels, executive and non-executive, considered by the priority and role to access private information. We generate an executive flag for each transaction, equal to 1 if that transaction is executed by an executive level and 0 otherwise.

### C. Summary statistics

The summary statistics of the all variables are tabulated in Table 1 for purchases and sales separately. Table 1 shows the different sign of CARs in the pre and post events for both purchase and sale transactions. This is consistent with findings by Seyhun [43] that the insiders are more likely to sell (purchase) shares following periods of significant price appreciation (declines). Our findings are also consistent with the insiders trading in anticipation of subsequent price reversals documented in Rozeff and Zaman [44] who show that insiders predominantly buy (sell) shares in value (growth) firms and interpret this as evidence of insiders trading against the market’s over-reaction to the past performance. Such trading behavior is consistent with insiders purchasing (selling) securities with high (low) expected returns or the greatest amount of undervaluation (overvaluation) [45, 46]. Further, we find the average insider profit is positive for purchases and negative for sales suggesting that the insiders successfully predict the direction of future returns. This implies that the insiders tend to be contrarian traders, buying after a fall and selling at the back of a rise, thus is another indicator that they are better informed than outside investors.

**Table 1:** Summary statistics.

Panel A: Summary statistics									
Purchase Obs = 3,549					Sale Obs = 2,732				
Variables		Mean	Median	Std. Dev	Variables	Mean	Median	Std. Dev.	
CAR(-250,-1)		-0.02506	0.00741	1.12704	CAR(-250,-1)	0.05294	-0.02196	1.20433	
CAR(1,20)		0.00670	0.06416	0.12780	CAR(1,20)	-0.03580	0.11830	0.14944	
AT proxy1		0.00026	0.00004	0.04592	AT proxy1	-0.00222	-0.00012	0.03417	
AT proxy2		0.01293	0.00000	0.68751	AT proxy2	-0.00430	-0.00054	0.58168	
Size		8.60398	8.33485	1.90211	Size	8.82096	8.54384	1.89990	
BM		1.15809	0.69500	2.24610	BM	1.03224	0.60804	1.99344	
ROA		0.28765	0.04466	0.20362	ROA	0.27929	0.27522	0.20269	
LEV		0.04694	0.29364	0.07998	LEV	0.04463	0.04524	0.08730	
TS		8.58449	8.70549	1.26534	TS	8.81638	8.85708	1.10729	
**Purchase**									
Variables	CAR(1,20)	CAR(-250,-1)	AT proxy1	AT proxy2	Size	BM	ROA	LEV	TS
CAR(1,20)	1	-0.0774***	-0.0547***	-0.0488***	0.0155	-0.0211	-0.0176	-0.0016	-0.0288*
CAR(-250,-1)	-0.079***	1	-0.0102	-0.0059	-0.0173	-0.0307*	0.0535***	-0.0035	-0.0397*
AT proxy1	0.012	0.004	1	0.7002***	-0.0375*	0.0327*	-0.0055	-0.0145	-0.0699***
AT proxy2	0.016	0.005	0.519***	1	-0.0203	0.0266	-0.0165	-0.0288*	-0.0258
Size	0.021	0.007	-0.023	-0.030*	1	-0.5106***	0.1679***	0.0993***	0.0303*
BM	-0.027	0.013	0.083***	0.075***	-0.353***	1	-0.2572***	0.0132	-0.0644***
ROA	-0.008	0.075***	0.003	0.009	0.122***	-0.018	1	-0.3222***	-0.1835***
LEV	-0.004	-0.002	-0.014	-0.004	0.085***	-0.003	-0.275***	1	0.1786***
TS	-0.028*	0.005	-0.040**	-0.027	0.048***	-0.188***	-0.181***	0.185***	1
**Sale**									
Variables	CAR(1,20)	CAR(-250,-1)	AT proxy1	AT proxy2	Size	BM	ROA	LEV	TS
CAR(1,20)	1	-0.0929***	-0.0387**	-0.0319	0.0507***	-0.0201	-0.0277	0.0288	-0.0449**
CAR(-250,-1)	-0.093***	1	-0.0428**	-0.0268	-0.0386**	-0.0677***	-0.0024	0.023	0.0557***
AT proxy1	0.001	-0.03	1	0.7065***	0.093***	-0.0253	-0.0064	0.0227	-0.0976***
AT proxy2	0.01	-0.007	0.573***	1	0.0609***	-0.0076	-0.0127	0.0308	-0.0478*
Size	0.078***	-0.009	0.076***	0.033*	1	-0.3982***	0.1465***	0.0912***	-0.0359*
BM	-0.042**	-0.029	-0.091***	-0.070***	-0.310***	1	-0.1861***	0.0492**	-0.0108
ROA	-0.045**	0.040**	0.022	0.031*	0.128***	0.004	1	-0.2704***	-0.2489***
LEV	0.022	0.015	0.012	-0.009	0.067***	0.024	-0.242***	1	0.2188***
TS	-0.055***	0.02	0.006	-0.002	-0.013	-0.225***	-0.224***	0.214***	1

This table presents descriptive statistics for all variables in this study. CAR (1,20) is a monthly average cumulative abnormal return over the window (1,20) after trading day. CAR (-250,-1) is a monthly average cumulative abnormal return over the one year window prior trading day. AT Proxy1 is a monthly change in AT calculated by stock-day level of number of ordered message and trade value in two adjacent months. AT Proxy2 is a monthly change in AT calculated by stock-day level of total ordered value and total traded value in two adjacent months. Size is firm size. BM is book to market ratio. ROA is return on assets, measured as net income divided by total asset at previous year end. LEV

is leverage level. TS is signed trade size.

*** p<0.01

** p<0.05

* p<0.1

Lower-triangular cells report Pearson’s correlation coefficients, upper-triangular cells are Spearman’s rank correlation

The correlation coefficients are presented in Panel B of Table 1. We provide two types of correlation analysis: i) Lower-triangular cells report Pearson’s correlation coefficients and ii), Upper-triangular cells are Spearman’s rank correlation. The result of Pearson’s correlation shows that there is no correlation between AT proxy and the cumulative abnormal returns in both proxies. However, the Spearman’s rank correlation shows correlation between AT Proxy1, AT Proxy2 and CAR (-250,-1) in both purchase and sale transactions. Trade size is negatively correlated with both proxies implying that the higher the AT intensity, the smaller the trade size. Our findings are consistent with the work by Aitken (2014) that AT speed can encompass thousands of trades in a second. This reflects higher trade counts along with smaller trade sizes.

## Empirical results

### A. Baseline regressions

To investigate the impact of AT on the insider trading performance, we estimate the following regression model:

$${CAR}_{i,t}=\alpha +\beta {AT\;proxy}_{i,t}+\gamma {controls}_{i,t}+FirmFE+YearFE+\varepsilon_{i,t}$$ (6)

where CARi,t is the cumulative abnormal return over 20 days, AT proxy is as described above. The control variables are firm size, book to market ratio, leverage, ROA and trade size.

Table 2 presents results from our baseline regressions with industry-year fixed effects and firm level clustered standard error. The coefficient estimates for both AT proxies are insignificant. Hence, we fail to reject the null hypothesis (H1), indicating that there is no association between both AT proxies and insider trading profits in both purchases and sales. The control variables are significant in the right direction, e.g., firm size and ROA. The coefficient on the cumulative abnormal return over one-year period is negative significant, capturing return momentum before and after insider transaction [47]. As a robustness check, we also compute the cumulative return over one week and 3 months windows (e.g. CAR(1,5) and CAR(1,60)) as proxies for our insider trading profits. Overall, results are robust to different event windows.

**Table 2:** Baseline regression.

	ATProxy1						ATProxy2					
	CAR(1,5)		CAR(1,20)		CAR(1,60)		CAR(1,5)		CAR(1,20)		CAR(1,60)	
	Buy	Sell	Buy	Sell	Buy	Sell	Buy	Sell	Buy	Sell	Buy	Sell
AT	-0.00273	-0.0717	0.03	-0.0819	0.158	-0.174	-0.000237	-0.000358	0.00355	0.00356	0.00575	0.00874
	-0.0139	-0.0512	-0.0451	-0.118	-0.108	-0.243	-0.000937	-0.00264	-0.0022	-0.00671	-0.00617	-0.0138
CAR(-250,-1)	-0.000995	-0.00116	-0.00736***	-0.00965***	-0.0187**	-0.0171*	-0.000996	-0.00113	-0.00736***	-0.00962***	-0.0186**	-0.0170*
	-0.00112	-0.00129	-0.00279	-0.00343	-0.00748	-0.00929	-0.00112	-0.00129	-0.00279	-0.00342	-0.00749	-0.00928
size	0.00224	0.00103	0.0164	0.0187*	0.0151	0.0457*	0.00224	0.000826	0.0164	0.0185*	0.0151	0.0453*
	-0.00366	-0.00415	-0.0103	-0.0104	-0.021	-0.0258	-0.00366	-0.00415	-0.0103	-0.0104	-0.021	-0.0258
bm	9.37E-05	-0.00082	0.00532*	-0.000129	0.00441	0.00492	9.47E-05	-0.000688	0.00528	0.000198	0.00453	0.00567
	-0.00133	-0.00159	-0.00322	-0.00319	-0.00763	-0.00738	-0.00133	-0.00162	-0.00323	-0.00318	-0.00763	-0.00754
roa1	0.0074	-0.0299	-0.0881	-0.185**	-0.111	-0.554***	0.00745	-0.0303	-0.0886	-0.186**	-0.115	-0.555***
	-0.0178	-0.0234	-0.0594	-0.0908	-0.169	-0.2	-0.0178	-0.0234	-0.0594	-0.0906	-0.168	-0.199
lev	0.00165	0.0218	-0.00128	-0.00513	0.111	-0.148	0.00168	0.0219	-0.00172	-0.00475	0.11	-0.147
	-0.0191	-0.0219	-0.0487	-0.0527	-0.103	-0.124	-0.0191	-0.0219	-0.0487	-0.0526	-0.103	-0.124
ts	0.00386*	0.000249	0.00762	-0.00527	0.0062	-0.0402**	0.00386*	0.00058	0.00761	-0.00453	0.00545	-0.0385*
	-0.00222	-0.00297	-0.00513	-0.00875	-0.0122	-0.0201	-0.00221	-0.003	-0.00514	-0.00877	-0.0123	-0.0203
Constant	-0.0522	-0.0448	-0.224**	-0.187*	-0.275	-0.137	-0.0522	-0.046	-0.224**	-0.192*	-0.268	-0.15
	-0.0388	-0.0393	-0.0993	-0.113	-0.221	-0.269	-0.0387	-0.0399	-0.0995	-0.112	-0.221	-0.27
Industry FE	Yes	Yes	Yes	Yes	Yes	Yes	Yes	Yes	Yes	Yes	Yes	Yes
Year FE	Yes	Yes	Yes	Yes	Yes	Yes	Yes	Yes	Yes	Yes	Yes	Yes
Observations	3,327	2,665	3,327	2,665	3,327	2,665	3,327	2,665	3,327	2,665	3,327	2,665
R-squared	0.005	0.01	0.015	0.025	0.017	0.033	0.005	0.008	0.015	0.024	0.016	0.033

Table 2 reports results for AT effect on insider trading profits for all Thai stocks over the period of 2010 to 2016. The dependent variable represents insider trading profits, CAR (1,5), CAR (1,20) and CAR (1,60) measured as a monthly average cumulative abnormal return over the window (1,5), (1,20) and (1, 60), respectively. The independent variable is AT Proxy1 and AT Proxy2. AT Proxy1 is a monthly change in AT calculated by stock-day level of number of ordered message and trade value in two adjacent months. AT Proxy2 is a monthly change in AT calculated by stock-day level of total ordered value and total traded value in two adjacent months. The control variables included in the regression are listed as follows: CAR (-250,-1) is a monthly average cumulative abnormal return over the one year window prior trading day. Size is firm size. BM is book to market ratio. ROA is return on assets, measured as net income divided by total asset at previous year end. LEV is leverage level. TS is signed trade size. Industry and year fixed effects are also included. T-statistics (reported in parentheses) are computed based on standard errors clustered at the firm level.

***, **, and * indicate significance at the 1%, 5%, and 10% levels, respectively.

### B. Effect of trade size on the relationship between AT and insider trading profits

To test our second hypothesis, we separate sample by trade size into 3 portfolios, perform regression analysis for all portfolios, purchase and sale separately and report the results in Table 3. Panel A of Table 3 reports insider purchase transactions, indicating insignificant coefficient for both AT proxies. Hence, for the purchase transactions, we are unable to reject the null hypothesis. As noted by Lakonishok and Lee [25] and Jeng et al. [26], the buy trades of insiders are driven by privy information, not public information. As such the advantage of AT in processing public information at ultra-high speed has no impact on insider purchase transactions.

**Table 3:** AT and insider by trade size.

Panel A: Purchase transactions in different trade size portfolio						
	ATProxy1			ATProxy2		
	Size S	Size M	Size L	Size S	Size M	Size L
AT	0.0331	-0.0158	0.26	0.00414	0.00428	0.0111
	-0.0452	-0.129	-0.201	-0.00236	-0.00802	-0.0115
CAR(-250,-1)	4.94E-05	-0.0124**	-0.00091	0.000163	-0.0125**	-0.00097
	-0.00579	-0.00522	-0.00551	-0.00579	-0.00522	-0.00553
size	0.0394**	0.0283	-0.0144	0.0394**	0.0286	-0.0153
	-0.016	-0.0222	-0.0172	-0.016	-0.0222	-0.0172
bm	0.0113***	-8.37E-06	-0.00667	0.0113***	0.000382	-0.00678
	-0.00406	-0.0161	-0.00702	-0.00407	-0.0159	-0.00704
roa1	-0.174	-0.177	-0.0329	-0.178	-0.174	-0.0342
	-0.159	-0.126	-0.0916	-0.16	-0.129	-0.0914
lev	-0.0815	-0.0279	0.0822	-0.0827	-0.029	0.0766
	-0.0916	-0.0851	-0.0999	-0.0919	-0.0845	-0.0959
ts	0.0102	0.0311	-0.00201	0.0101	0.032	-0.00175
	-0.00989	-0.0211	-0.0167	-0.00999	-0.0212	-0.017
Constant	-0.432***	-0.517*	0.132	-0.431***	-0.528*	0.138
	-0.128	-0.289	-0.243	-0.129	-0.291	-0.247
Industry FE	Yes	Yes	Yes	Yes	Yes	Yes
Year FE	Yes	Yes	Yes	Yes	Yes	Yes
Observations	1,219	1,071	1,037	1,219	1,071	1,037
R-squared	0.05	0.055	0.015	0.052	0.055	0.014
Panel B: Sale transactions in different trade size portfolio						
	ATProxy1			ATProxy2		
	Size S	Size M	Size L	Size S	Size M	Size L
AT	-0.158	-0.113	0.615*	-0.00254	0.0027	0.0508***
	-0.167	-0.17	-0.323	-0.00804	-0.00968	-0.0188
CAR(-250,-1)	-0.0116	-0.0109*	-0.00302	-0.0114	-0.0108*	-0.00299
	-0.00722	-0.00636	-0.00458	-0.00711	-0.00637	-0.00469
size	-0.00784	0.0244	0.0194	-0.00888	0.0238	0.0236
	-0.0179	-0.022	-0.025	-0.0182	-0.022	-0.0249
bm	-0.0113**	-0.0156	-0.00551	-0.0111**	-0.0158	-0.00464
	-0.00519	-0.013	-0.0169	-0.00535	-0.013	-0.0169
roa1	-0.138	-0.314	-0.248***	-0.143	-0.315	-0.255***
	-0.147	-0.256	-0.0948	-0.145	-0.255	-0.0917
lev	0.0214	-0.0588	-0.07	0.0213	-0.0593	-0.102
	-0.102	-0.0703	-0.138	-0.103	-0.0704	-0.132
ts	0.0242	0.0483	-0.0228	0.0262	0.0473	-0.0181
	-0.0225	-0.0296	-0.0224	-0.0237	-0.0299	-0.0234
Constant	-0.217	-0.627*	-0.0126	-0.221	-0.615*	-0.0916
	-0.22	-0.355	-0.297	-0.224	-0.358	-0.315
Industry FE	Yes	Yes	Yes	Yes	Yes	Yes
Year FE	Yes	Yes	Yes	Yes	Yes	Yes
Observations	785	917	963	785	917	963
R-squared	0.068	0.053	0.031	0.066	0.053	0.031

Table 3 reports results for AT effect on insider profits in a sample from 2010 to 2016 for all stocks in the Thai market. The dependent variable represents insider profits (CAR (1,20)), measured as a monthly average cumulative abnormal return over the window (1,20). The independent variable is AT Proxy1 and AT Proxy2. AT Proxy1 is a monthly change in AT calculated by stock-day level of number of ordered message and trade value in two adjacent months. AT Proxy2 is a monthly change in AT calculated by stock-day level of total ordered value and total traded value in two adjacent months. The control variables included in the regression are listed as follows: CAR (-250,-1) is a monthly average cumulative abnormal return over the one year window prior trading day. Size is firm size. BM is book to market ratio. ROA is return on assets, measured as net income divided by total asset at previous year end. LEV is leverage level. TS is signed trade size. Size S, Size M and Size L are the portfolio of stocks being sorted into terciles by their trade size. The resultant number of observations is different in the terciles because the sorting is by trade-size. Panel A presents the regression analysis of purchase transactions in different trade size portfolio and Panel B presents the regression analysis of sale transactions in different trade size portfolio. Industry and year fixed effects are also included. T-statistics (reported in parentheses) are computed based on standard errors clustered at the firm level.

***, **, and * indicate significance at the 1%, 5%, and 10% levels, respectively.

Panel B of Table 3 reports results for the sale transaction. In the presence of all control variables, the result in sale transaction for large trade size has positive and significant coefficient on both AT proxies suggesting that increased AT reduces the insider sale profitability. One unit increased in AT results in a drop of insider sale profit 0.62% and 0.051% for each AT proxy respectively. These findings support the asymmetric information hypothesis, demonstrating that AT reduces insider trading profits. Further, consistent with O’Hara et al. [39], we are able to reject our second hypothesis such that large trade size benefits AT performance, resulting in lower insider trading profits. AT has advantage over the corporate insider when trade size is large. Corporate insiders often use large trade size, causing more pronounced price impact. However, with ultra-high speed, AT can trade a more significant amount with less price impact. Hence, the presence of AT improves information efficiency and lower insider trading profits. In addition, our findings from both panels of Table 3 are consistent with the difference in trading motivations between corporate insider purchases and sales. The motivation for the insider purchases is typically private information [25, 26] while sales can also occur for public information or non-information reasons such as liquidity and hedging [27, 28].

### C. Can AT beat non-executive insider?

Executive and non-executive directors access to private and price-sensitive information differently. Executive management have priority to access data and gather all information from all departments. To test our third hypothesis, we conduct regression analysis for purchase and sale transactions separately for both executive and non-executive insiders. For insider purchase transactions, all panels of Table 4 indicate insignificant coefficient for both AT proxies. We fail to reject the third hypothesis for the insider purchase transactions. Again, consistent with Lakonishok and Lee [25] and Jeng et al. [26], we find insider purchase transactions are likely motivated by private information that cannot be detected by the outsiders. Hence, when it comes to purchasing stocks, algorithmic traders with their superior public information cannot beat corporate insiders with private information.

**Table 4:** AT and insider’s management level.

**Panel A: Executive and Non-Executive full samples**												
	Executive						Non-Executive					
	ATProxy1			ATProxy2			ATProxy1			ATProxy2		
	Buy		Sell	Buy		Sell	Buy		Sell	Buy		Sell
AT	0.00198		-0.340**	0.00422		-0.00883	0.0501		0.0859	0.00109		0.0161*
	-0.0719		-0.134	-0.00327		-0.00539	-0.0778		-0.176	-0.00555		-0.00952
Constant	-0.202*		-0.131	-0.206*		-0.151	-0.177		-0.0167	-0.175		-0.0396
	-0.112		-0.166	-0.113		-0.166	-0.184		-0.176	-0.185		-0.177
Control Variables	Yes		Yes	Yes		Yes	Yes		Yes	Yes		Yes
Industry FE	Yes		Yes	Yes		Yes	Yes		Yes	Yes		Yes
Year FE	Yes		Yes	Yes		Yes	Yes		Yes	Yes		Yes
Observations	1,959		1,507	1,959		1,507	1,368		1,158	1,368		1,158
R-squared	0.011		0.033	0.012		0.03	0.033		0.02	0.033		0.024
**Panel B: Executive and Non-Executive by Trade Size for ATProxy1**												
	Executive						Non-Executive					
	Purchase			Sale			Purchase			Sale		
	SizeS	SizeM	SizeL	SizeS	SizeM	SizeL	SizeS	SizeM	SizeL	SizeS	SizeM	SizeL
AT	0.00842	0.0124	0.248	-0.503**	-0.262*	0.489	0.0500	0.0854	0.305	-0.0568	-0.187	0.535*
	(0.0667)	(0.206)	(0.202)	(0.218)	(0.146)	(0.699)	(0.0674)	(0.132)	(0.518)	(0.168)	(0.344)	(0.0229)
Constant	-0.290	-0.618	-0.561**	-0.261	-0.518	0.552	-0.447	-1.223**	1.327***	-0.0724	-0.797	0.133
	(0.213)	(0.426)	(0.262)	(0.481)	(0.395)	(0.467)	(0.338)	(0.488)	(0.489)	(0.350)	(0.627)	(0.453)
Control Variables	Yes	Yes	Yes	Yes	Yes	Yes	Yes	Yes	Yes	Yes	Yes	Yes
Industry FE	Yes	Yes	Yes	Yes	Yes	Yes	Yes	Yes	Yes	Yes	Yes	Yes
Year FE	Yes	Yes	Yes	Yes	Yes	Yes	Yes	Yes	Yes	Yes	Yes	Yes
Observations	665	645	649	376	548	583	554	426	388	409	369	380
R-squared	0.054	0.058	0.028	0.149	0.065	0.038	0.078	0.132	0.074	0.067	0.078	0.064
**Panel C: Executive and Non-Executive by Trade Size for ATProxy2**												
	Executive						Non-Executive					
	Purchase			Sale			Purchase			Sale		
	SizeS	SizeM	SizeL	SizeS	SizeM	SizeL	SizeS	SizeM	SizeL	SizeS	SizeM	SizeL
AT	0.00557	0.00988	0.00487	-0.00684	-0.00534	-0.0628	-0.000250	0.00248	0.00256	0.00256	0.00296	0.0663***
	(0.00451)	(0.0116)	(0.00671)	(0.00795)	(0.00834)	(0.0768)	(0.00486)	(0.0124)	(0.0661)	(0.00505)	(0.0442)	(0.0229)
Constant	-0.290	-0.636	-0.559**	-0.334	-0.513	0.545	-0.443	-1.221**	1.213**	-0.0960	-0.734	-0.0790
	(0.212)	(0.425)	(0.266)	(0.519)	(0.406)	(0.468)	(0.339)	(0.487)	(0.489)	(0.353)	(0.651)	(0.450)
Control Variables	Yes	Yes	Yes	Yes	Yes	Yes	Yes	Yes	Yes	Yes	Yes	Yes
Industry FE	Yes	Yes	Yes	Yes	Yes	Yes	Yes	Yes	Yes	Yes	Yes	Yes
Year FE	Yes	Yes	Yes	Yes	Yes	Yes	Yes	Yes	Yes	Yes	Yes	Yes
Observations	665	645	649	376	548	583	554	426	388	409	369	380
R-squared	0.057	0.059	0.026	0.135	0.062	0.037	0.077	0.131	0.088	0.067	0.076	0.073
Number of firmid	127	149	123	112	162	118	129	113	93	98	126	104

Table 4 reports results for AT effect on insider profits in a sample from 2010 to 2016 for all stocks in the Thai market. The dependent variable represents insider profits (CAR (1,20)), measured as a monthly average cumulative abnormal return over the window (1,20) after trading day. The independent variable is AT Proxy1 and AT Proxy2. AT Proxy1 is a monthly change in AT calculated by stock-day level of number of ordered message and trade value in two adjacent months. AT Proxy2 is a monthly change in AT calculated by stock-day level of total ordered value and total traded value in two adjacent months. The control variables included in the regression are listed as follows: CAR (-250,-1) is a monthly average cumulative abnormal return over the one year window prior trading day. Size is firm size. BM is book to market ratio. ROA is return on assets, measured as net income divided by total asset at previous year end. LEV is leverage level. TS is signed trade size. Size S, Size M and Size L are the portfolio of stocks being sorted into terciles by their trade size. Results for Executive and Non-executive level are reported separately and split by purchase and sale transactions. Industry and year fixed effects are also included. T-statistics (reported in parentheses) are computed based on standard errors clustered at the firm level.

***, **, and * indicate significance at the 1%, 5%, and 10% levels, respectively.

In addition, Panel A of Table 4 indicates significant and negative (positive) coefficient estimate for AT Proxy1 (AT Proxy2) in the case of executive (non-executive) sale transactions. This implies that AT enhances (reduces) executives (non-executives) profits on their sale transactions. Hence, for the insider sale transactions, we are able to reject our third hypothesis and find support to the information hierarchy hypothesis such that executives are true insiders equipped with better access to private information than non-executives do. Findings on executives support attentive insider trading hypothesis which states that corporate insiders are among the most attentive traders of their own stocks and easily recognize profitable trading opportunities when they observe public information related to their firm. That is, AT cannot beat well-informed traders (i.e. executive traders) but rather enhance their trading profits in the sale transactions. Executives benefit from liquidity and public information efficiency induced by AT and make larger profits.

Further, our findings on non-executive sale transactions support the asymmetric information hypothesis, indicating the higher the information asymmetry, the higher the insider trading profits. Consistent with To et al. [14], we find not all insiders are on the inside, non-executive directors appear to be less informative and their trading profits decrease in the presence of AT who has superior public information. Hence, AT can beat non-executive sales.

Finally, Panels B and C of Table 4 show AT cannot beat executive sales such that executive profits more with the increase of AT activities, for small and medium trade size. Seyhun [23] and Ravina and Sapienza [48] also find that top executives outperform other groups of insiders in share trading, most likely due to their privileged position with respect to the corporate information set. Again, we find AT beat non-executive sales with large trade size. This confirm our previous findings that with ultra-high speed information processing, AT can trade large transaction size with less price impact and non-executive sales are less informative and likely to trade for non-informational reason. Hence, AT reduces information asymmetry and non-executive trading profits.

### D. Robustness checks

We run a battery of robustness checks. First, we use various event windows in computing insider trading profits. Our results reported in Table 2 are robust to different event windows. Second, we adopt two proxies in constructing algorithmic trading and in general, results for both proxies are relatively similar. Finally, we employ 2SLS instrumental-variable (IV) regression in order to alleviate endogeneity concerns using an industry median of AT as an instrument variable as shown in Table 5. The idea for chosen instrument variable is that it is unlikely that AT in one firm could have any influence over AT in other firms. In other words, any variation at industry level is beyond the control of a firm because there are many firms in an industry. As such, each firm takes any change at the industry level as given. An industry median is therefore likely to be exogenous. Hence, both the relevance and exclusion requirements for an instrumental variable should be satisfied. Findings reported in Table 5, in general, confirms our previous findings that AT enhances executive sales and that when it comes to purchasing stocks, algorithmic traders with their superior public information cannot beat corporate insiders with private information.

**Table 5:** AT and insider’s management level– 2SLS instrumental variable approach.

Panel A: Executive	ATProxy1				ATProxy2			
	Purchase		Sale		Purchase		Sale	
	1st stage	2nd Stage	1st stage	2nd Stage	1st stage	2nd Stage	1st stage	2nd Stage
AT	0.815**		0.773***		0.755**		0.627***	
	-3.303		-6.385		-2.492		-2.81	
AT_predict		-0.301		-0.899*		0.013		-0.018
		-0.878		(-1.763)		-1.078		(-0.638)
car_n250n1m	0.001	-0.004	-0.001**	-0.010*	0.003	-0.004	-0.001	-0.009
	-0.836	(-0.919)	(-2.095)	(-1.814)	48	(-0.966)	(-0.124)	(-1.618)
size	0.002	0.021*	-0.003	0.005	0.029	0.019	-0.09	0.005
	-0.726	-1.659	(-0.999)	-0.401	-0.861	-1.54	(-1.278)	-0.408
bm	0.002	0.010***	-0.006**	-0.011	0.027	0.009***	-0.124	-0.009
	-1.231	-3.286	(-2.085)	(-2.017)	-1.094	-2.942	(-1.547)	(-1.539)
roa1	-0.02	-0.12	-0.001	-0.171	0.117	-0.113	-0.001	-0.171
	(-1/057)	(-1.362)	(-0.093)	(-1.564)	-0.249	(-1.215)	(-0.004)	(-1.574)
lev	-0.001	-0.026	0.014	0.107	0.207	-0.025	-0.029	0.009
	-0.073	(-0.394)	-1.04	-0.247	-0.856	(-0.364)	(-0.271)	-0.125
ts	-0.009**	0.006	-0.007**	-0.008	-0.137**	0.011*	-0.063	-0.001
	(-2.344)	-0.915	(-2.081)	(-0.524)	(-2.061)	-1.788	(-0.979)	-0.047
Constant	0.070*	-0.182	0.100**	-0.077	0.966	-0.215*	-0.013	-1.854
	-1.868	(-1.557)	-0.127	(-0.441)	-1.437	(-1.893)	(-0.230)	0.027
Observations	1,959	1,959	1,507	1,507	1,959	1,959	1,507	1,507
R-squared	0.073	0.012	0.133	0.032	0.093	0.011	0.146	0.029
Panel B: Non-Executive	ATProxy1				ATProxy2			
	Purchase		Sale		Purchase		Sale	
	1st stage	2nd Stage	1st stage	2nd Stage	1st stage	2nd Stage	1st stage	2nd Stage
AT	1.160***		0.958***		0.791***		1.131***	
	-5.071		-5.874		-3.05		-7.285	
AT_predict	-0.03		0.821			-0.043	
	(-0.098)		-1.369	-1.379		(-1.163)		-2.934
car_n250n1m	0.001	-0.010**	0.001	-0.012**	0.02	-0.009**	0.006	-0.012**
	-1.123	(-2.351)	-1.014	(-2.213)	-0.967	(-2.096)	-0.568	(-2.200)
size	-0.003	0.017	0.007*	0.011	-0.081	0.014	0.019	0.014
	(-0.727)	-0.94	-1.705	-0.668	(-1.363)	-0.784	-0.551	-0.911
bm	0.002	0	-0.001	0	0.007	0.001	-0.034	0.001
	-0.985	-0.057	(-0.401)	(-0.033)	-0.209	-0.094	(-1.425)	-0.255
roa1	-0.028	-0.123	0	-0.093	-0.455	-0.1418	0.066	-0.097
	(-1.033)	(-1.613)	(-0.033)	(-0.729)	(-1.475)	(-1.795)	-0.285	(-0.759)
lev	0.007	-0.034	-0.017	0.007	-0.245	-0.043	0.014	-0.007
	-0.317	(-0.446)	(-1.045)	(—0.063)	(-1.225)	(-0.557)	-0.063	(-0.060)
ts	-0.001	0	-0.002	-0.016	0.105*	0.005	-0.150*	-0.009
	(-0.333)	-0.029	(-0.413)	(-1.656)	-1.89	-0.425	(-1.656)	(-0.630)
Constant	0.034	-0.173	-0.037	0.005	-0.221	-0.183	1.297	-0.101
	-0.798	(-0.937)	(-0.666)	-0.028	(-0.101)	(-0.976)	-1.428	(-0.569)
Observations	1,368	1,368	1,158	1,158	1,368	1,368	1,158	1,158
R-squared	0.085	0.033	0.14	0.024	0.026	0.033	0.18	0.03

Table 5 reports 2SLS IV results for AT effect on insider profits in a sample from 2010 to 2016 for all stocks in the Thai market. The dependent variable represents insider profits (CAR (1,20)), measured as a monthly average cumulative abnormal return over the window (1,20) after trading day. The independent variable is AT Proxy1 and AT Proxy2. AT Proxy1 is a monthly change in AT calculated by stock-day level of number of ordered message and trade value in two adjacent months. AT Proxy2 is a monthly change in AT calculated by stock-day level of total ordered value and total traded value in two adjacent months. The control variables included in the regression are listed as follows: CAR (-250,-1) is a monthly average cumulative abnormal return over the one year window prior trading day. Size is firm size. BM is book to market ratio. ROA is return on assets, measured as net income divided by total asset at previous year end. LEV is leverage level. TS is signed trade size. Results for Executive and Non-executive level are reported separately and split by purchase and sale transactions. Industry and year fixed effects are included. T-statistics (reported in parentheses) are computed based on standard errors clustered at the firm level.

***, **, and * indicate significance at the 1%, 5%, and 10% levels, respectively.

## Conclusions

The existence of insider trading profit deems to be inconsistent with the strong form of the efficient market model which states that all information, public and private, is fully reflected in stock prices. To make the market more efficient, ones need to capture the abnormal signal and react as soon as possible to keep the market unbiased informative. The algorithmic trading gains interests from financial market researchers. However, most studies in AT are in the developed markets where AT is a significant proportion of trading activities. Algorithmic trading has some characteristics supported by empirical evidences that AT actively monitors market conditions. This is consistent with the fact that AT has lower monitoring costs and faster speed to react the market condition. In this study, we find that AT can restrain insider’s profits. Specifically, we document an effect of AT on insider trading profits in a big trade size portfolio such that AT reduces insider profits on sale transactions. Furthermore, AT can beat non-executive trading on sale side but enhance executive sales. One promising area for future research from this study is to further investigate what governance roles are played by AT, or by other significant market structure changes, in other important corporate decisions that are made by management and insiders of firms.
